# Role of New Antifungal Agents in the Treatment of Invasive Fungal Infections in Transplant Recipients: Isavuconazole and New Posaconazole Formulations

**DOI:** 10.3390/jof1030345

**Published:** 2015-10-15

**Authors:** Julius Li, Cynthia T. Nguyen, Julia Garcia-Diaz

**Affiliations:** 1 Department of Pharmacy, Ochsner Medical Center, New Orleans, LA 70123, USA; E-Mails: juli@ochsner.org (J.L.); cynthia.nguyen@ochsner.org (C.T.N.); 2 Department of Infectious Diseases, Ochsner Medical Center, 1514 Jefferson Highway, New Orleans, LA 70123, USA

**Keywords:** antifungals, transplantation, candidiasis, aspergillosis

## Abstract

Invasive fungal infections are a major cause of morbidity and mortality among solid organ transplant (SOT) and hematopoietic stem cell transplant (HSCT) recipients. Transplant patients are at risk for such invasive fungal infections. The most common invasive fungal infections are invasive candidiasis in the SOT and invasive aspergillosis in the HSCT. In this article, we will discuss the epidemiology of invasive fungal infections in the transplant recipients and susceptibility patterns of the fungi associated with these infections. Additionally, the pharmacology and clinical efficacy of the new antifungal, isavuconazole, and the new posaconazole formulations will be reviewed. Isavuconazole is a new extended-spectrum triazole that was recently approved for the treatment of invasive aspergillosis and mucormycosis. Advantages of this triazole include the availability of a water-soluble intravenous formulation, excellent bioavailability of the oral formulation, and predictable pharmacokinetics in adults. Posaconazole, a broad-spectrum triazole antifungal agent, is approved for the prevention of invasive aspergillosis and candidiasis in addition to the treatment of oropharyngeal candidiasis. Posaconazole oral suspension solution has shown some limitations in the setting of fasting state absorption, elevated gastrointestinal pH, and increased motility. The newly approved delayed-release oral tablet and intravenous solution formulations provide additional treatment options by reducing interpatient variability and providing flexibility in these set of critically ill patients. This review will detail these most recent studies.

## 1. Introduction

Invasive fungal infections (IFI) are a major cause of morbidity and mortality among solid organ transplant (SOT) and hematopoietic stem cell transplant (HSCT) recipients. The number of transplants performed in the United States continues to rise with almost 30,000 SOT performed in 2013, and about 20,000 HSCT in the same year [1,2]. These IFI in the transplant population are difficult to treat and the antifungals currently available are limited.

In 2001, the Transplant Associated Infection Surveillance Network (TRANSNET), a consortium of 23 academic tertiary care medical centers throughout the United States, was established to perform prospective surveillance among transplant recipients with the purpose to better understand IFIs. Enrollment of SOT recipients occurred between March 2001 and September 2005 and the surveillance period for cases was March 2001 through 2006. A total of 16,808 SOT were enrolled and followed prospectively for the development of IFIs. A 12-month cumulative incidence rate of 3.1% (1.2% to 6.1%) was identified. The most common IFIs were invasive candidiasis (53%), invasive aspergillosis (19%), cryptococcosis (8%), non-*Aspergillus* molds (8%), endemic fungi (5%), and mucormycosis (2%) [3]. Similar data are reported by the Prospective Antifungal Therapy Alliance (PATH Alliance) which is comprised of 17 medical centers. A total of 429 adult SOT recipients with 515 IFIs were identified from March 2004 to September 2007. Most IFIs were caused by *Candida* spp. (59%), followed by *Aspergillus* spp. (24.8%), *Cryptococcus* spp. (7.0%), and other molds (5.8%). Invasive candidiasis was the most frequently observed IFI in all groups, except for lung recipients where invasive aspergillosis was the most common IFI [4].

A total of 16,200 HSCT were enrolled by TRANSNET and followed prospectively. An overall IFI incidence rate of 3.4% (0.9%–13.2%) was identified. Invasive aspergillosis comprised 43%, followed by invasive candidiasis (28%) and mucormycosis (8%). *A. fumigatus* was the most common *Aspergillus* species (44%) and *C. glabrata* (33%) was more common than *C. albicans* (20%) in contrast to SOT recipients. The majority of these patients had received allogeneic transplants (78%) and the remainder were autologous transplants (21%) [5].

A total of 915 invasive candidiasis were identified by TRANSNET and of those, 383 invasive *Candida* isolates were available for susceptibility testing. All isolates were susceptible to caspofungin. There was 10% resistance to fluconazole and this included 23% of *C. glabrata* isolates; *C. krusei* which is intrinsically resistance to fluconazole was excluded. However, resistance increased to 16% overall when *C. krusei* was added. *C. albicans*, *C. parapsilosis*, and *C. tropicalis* isolates exhibited only 1% resistance to fluconazole. Voriconazole resistance was 3% overall but was 8% among *C. glabrata* isolates. They also had posaconazole MIC values at or above the average achievable serum levels at 400 mg twice a day (oral suspension). All of the *C. krusei* isolates had voriconazole MIC values of <1 mcg/mL which is interpreted as fully susceptible [6].

The incidence of non-*Aspergillus* mold infections in the transplant population is also increasing. TRANSNET identified 169 infections in 169 patients (124 in HSCT and 45 in SOT); 105 Mucorales, 37 *Fusarium* spp., and 27 *Scedosporium* spp. The 90-day crude mortality rate was 56.6% [7].

Over the past decade, there has been a growth in the antifungal armamentarium which includes newer broad-spectrum azoles of which the most recent addition is isavuconazole; and new formulations of already available antifungals, posaconazole. Posaconazole (Noxafil^®^) is a triazole antifungal agent with an extended spectrum of antifungal activity. The efficacy and good tolerability of posaconazole oral suspension is well established. However, in order to overcome pharmacokinetic limits associated with the suspension, a new delayed-release tablet and intravenous (IV) solution were developed. This article reviews the pharmacokinetic properties of the new posaconazole formulations and also the efficacy data as it relates to the suspension. The pharmacokinetic advantages of the posaconazole delayed-release tablet compared with the suspension formulation include less interpatient variability, better systemic availability allowing for once-daily administration, and absorption that is unaffected by changes in gastric pH and they can be taken with or without food. The posaconazole IV solution provides an option for these same indications in patients who are unable to receive oral formulations.

The newest addition is isavuconazonium (Cresemba^®^); a broad spectrum prodrug of the triazole isavuconazole with efficacy against invasive fungal diseases including aspergillosis and mucormycosis. Some of its characteristics include linear dose-proportional pharmacokinetics, IV, and oral formulations allowing therapeutic streamlining, once daily dosing, absence of nephrotoxic solubilizing agents and excellent oral bioavailability independent of meal status and gastric acidity.

Both agents, posaconazole and isavuconazole are active against non-*C. albicans* spp., *Aspergillus* spp. and non-*Aspergillus* molds. In this article, we will review the data that is currently available for the new formulations of posaconazole and the newly approved azole, isavuconazole.

## 2. Chemistry of Posaconazole and Isavuconazole

Similar to other triazole antifungals, both posaconazole and isavuconazole inhibit lanosterol 14 α-demethylase enzyme, which prevents the conversion of lanosterol to ergosterol in the fungal cell membrane [8,9]. Inhibition of this process results in altered fungal cell membrane function and accumulation of toxic precursors and, thus, inhibition of growth and cell death. Both posaconazole and isavuconazole have enhanced activity against fungi resistant to other triazole antifungals due to side chain alterations [8,9].

Similar to intravenous (IV) voriconazole, IV posaconazole is formulated with sulfobutyl-ether-β-cyclodextrin (SBECD) due to its relatively poor aqueous solubility. Animal studies have demonstrated that accumulation of SBECD is associated with reversible renal tubular vacuolation in rats and foamy macrophages in dogs, but these studies utilized exposures higher (50-fold greater) than the ones used in clinical practice in humans. [10] Since SBECD is renally eliminated, its accumulation occurs in humans with renal dysfunction [10]. Due to these concerns, the manufacturer recommends to consider alternative options to IV posaconazole in patients with moderate to severe renal impairment (creatinine clearance <50 mL/min), unless a risk-benefit assessment justifies its use [11]. However, several studies of SBECD in humans with renal dysfunction including patients with acute kidney injury, and patients on renal replacement, have not validated these concerns; accumulation of SBECD in these subjects did not result in further insult to the kidneys or additional toxicities [10,12,13,14].

Isavuconazonium, the prodrug of isavuconazole, is highly water-soluble, eliminating the need for a solubilizing agent [15]. Isavuconazonium, rapidly and completely (>99%), undergoes enzymatic activation in the plasma into the active drug, isavuconazole, and an inactive cleavage product [16,17]. Exposure to the inactive cleavage product is negligible (~1%) compared to isavuconazole after oral or IV administration. Unlike the IV formulations of voriconazole and posaconazole, isavuconazonium does not require the addition of SBECD to facilitate solubility, thus eliminating concerns about the potential for nephrotoxicity due to accumulation of SBECD in renal dysfunction.

## 3. Pharmacokinetics and Pharmacodynamics

### 3.1. Posaconazole

Previously, posaconazole was only available as an oral suspension. The FDA approved delayed-release tablets and IV formulations in November 2013 and March 2014, respectively. The pharmacokinetic properties of the three posaconazole formulations are outlined in Table 1. Aside from differences in absorption, the majority of the pharmacokinetic parameters remain similar between the various posaconazole formulations. All three formulations are widely distributed, highly protein bound (>98%), and have a relatively long half-life (20–66 h) [18,19,20,21,22]. Additionally, although posaconazole is a strong CYP3A4 inhibitor, posaconazole primarily undergoes glucuronidation by uridine 5′-diphosphate (UDP)-glucuronosyltransferase enzymes. Consequently, posaconazole serum concentrations are largely unaffected by medications that inhibit or enhance the cytochrome P450 (CYP450) system [11].

Posaconazole oral suspension displays variable and inconsistent absorption. Absorption of the suspension is heavily reliant on a high-fat state, with fourfold greater bioavailability if administered with a high-fat meal compared to a fasting state. Absorption is also affected by medications that raise gastric pH and increase gastrointestinal motility [23]. These interactions are further described in the drug-drug interactions section. Posaconazole exposure is greater with split doses rather than as a single daily dose [24]. A rising multiple-dose ranging study among 103 healthy adult subjects, demonstrated saturation of absorption occurring with posaconazole doses above 800 mg. For doses between 50 mg to 800 mg, mean plasma posaconazole concentrations increased proportionally, however no further increases in plasma concentrations were observed with the 1200 mg dose. This characteristic has been attributed to the poor solubility of the posaconazole suspension [18].

Posaconazole delayed-release tablets offer the advantage of reliable absorption. The posaconazole tablets are developed in a pH-sensitive polymer matrix which is designed to inhibit the release of posaconazole until the drug reaches the elevated-pH environment of the small intestine, maximizing systemic absorption. Under fasting and fed conditions, the posaconazole delayed-release tablet maintains bioavailability [19]. Absorption of the delayed-release tablets is also reliable regardless of administration with food; however, the manufacturer still recommends taking the tablets with food. Unfortunately, the delayed-release tablet cannot be crushed or chewed as this may damage the integrity of the pH-sensitive matrix and, thus, the pharmacokinetics may be altered; this may limit its use among patients who cannot take medications orally and enteral administration is preferred [11].

**Table 1:** Pharmacokinetics for oral and intravenous posaconazole and isavuconazole formulations.

Drug	Posaconazole			Isavuconazole	
Dosage form	Delayed-release tablets (100 mg)	Intravenous (300 mg per vial)	Oral suspension (40 mg/mL, 105 mL)	Capsules (186 mg) ^#^	Intravenous (372 mg per vial) ^#^
Dosing	Load: 300 mg PO every 12 h for 24 h Maintenance: 300 mg PO daily	Load: 300 mg IV every 12 h for 24 h Maintenance: 300 mg IV daily	Prophylaxis: 200 mg PO every 8 h Treatment: 800 mg PO per day in divided doses	Load: 372 mg PO every 8 h for 6 doses (48 h) ^#^ Maintenance: 372 mg PO daily ^#^	Load: 372 mg IV every 8 h for 6 doses (48 h) ^#^ Maintenance: 372 mg IV daily ^#^
Administration	Delayed-release tablets should be swallowed whole, and not divided, crushed, or chewed	Infuse over 90 min via central venous line through an in-line filter. Infusion through a peripheral line should only be used as a one-time infusion over 30 min to avoid infusion-site reactions with multiple infusions	Oral suspension should be administered with a full meal, nutritional supplement, or acidic carbonated beverage	Capsules should be swallowed whole, and not divided, crushed, or chewed	Infuse over a minimum of 60 min via central venous line through an in-line filter. Avoid unnecessary vibration of vigorous shaking of solution to avoid formation of particulates
Bioavailability	54%	NA	Variable (8%–47%)	98%	NA
Effect of food	Unknown	NA	High-fat meal increases AUC and Cmax by 4-times compared to the fasting state	None, can be taken with or without food	NA
Time to peak concentration	4–5 h	1–2 h	3–5 h	3 h	End of infusion
Vd (mean)	226–295 L			450 L	
CSF penetration	Unknown			Very low to undetectable	
Protein binding	>98%			>99%	
Metabolism	Primarily metabolized via UDP glucuronidation P-glycoprotein efflux substrate			CYP3A4 and CYP3A5	
Half-life (mean)	26–31 h	27 h	20–66 h	130 h	
Excretion	71% eliminated via feces (66% as parent compound) 13% renally eliminated			46% eliminated in the feces, 46% renally eliminated (<1% as parent compound)	

PO = oral, IV = intravenous, NA = not available, Vd = volume of distribution, CSF = cerebrospinal fluid, ^#^ Administered as isavuconazonium sulfate (186 mg of isavuconazonium sulfate = 100 mg isavuconazole).

### 3.2. Isavuconazole

The pharmacokinetic properties of isavuconazole are also outlined in Table 1. Isavuconazole exhibits linear pharmacokinetics as demonstrated by proportional increases in Cmax and area under the concentration-time (AUC) curves following increases in dose [16]. Significant accumulation, up to five-fold, was observed in multiple-dose studies with both oral and IV formulations [17]. Further accumulation may be anticipated since isavuconazole exhibits a long half-life of approximately 130 h, and steady-state was likely not achieved in this study that sampled serum concentrations up to 14 days of therapy.

Studies in healthy volunteers and neutropenic patients demonstrate extensive bioavailability (98%) of isavuconazole, potentially allowing for a seamless transition from IV to oral therapy. No significant food effect was observed when given to healthy male volunteers in either a fasted or fed state. In addition to a long half-life, isavuconazole is highly protein bound (>99%) with a large volume of distribution (400–500 L). Isavuconazole is primarily metabolized by the CYP450 system, namely by the isoenzyme CYP3A4, which raises the concern of potential significant interactions with drugs metabolized through this pathway. Additionally, systemic clearance of isavuconazole was decreased in healthy subjects with moderate liver impairment suggesting the need for dose reductions in patients with underlying liver disease; however there are no recommended adjustments at this time [25].

## 4. Microbiology

Both posaconazole and isavuconazole demonstrate broader spectrum of antifungal activity compared to other triazoles. Notably, both agents exhibit fungistatic activity against many *Candida* spp. (including *C. glabrata* and *C. krusei*), and fungicidal activity against *Aspergillus* spp. [26,27,28,29]. The MIC50 and MIC90 for both agents against common fungi are listed in Table 2.

**Table 2:** Posaconazole MIC50 and MIC90 for common fungi. [26,27,28,29].

Fungus	Posaconazole		Isavuconazole	
	MIC_50_	MIC_90_	MIC_50_ Range	MIC_90_ Range
*Candida* spp.				
*C. albicans*	0.03	0.13	<0.002–0.03	<0.002–0.03
*C. glabrata*	1	2	0.25–2	0.5–8
*C. parapsilosis*	0.03	0.13	<0.015–0.03	0.03–0.125
*C. tropicalis*	0.06	1	<0.015–0.03	0.03–0.125
*C. krusei*	0.25	0.5	0.125–0.5	0.25–1
*C. lusitaniae*	0.03	0.13	-	-
*C. dubliniensis*	0.03	0.06	-	-
*Cryptococcus neoformans*	0.03	0.06	0.004–0.03	0.016–0.125
*Cryptococcus gattii*	0.03	0.125	0.03–0.32	0.06–0.125
*Aspergillus* spp.				
*A. fumigatus*	0.125	0.5	0.25–1	0.5–2
*A. flavus*	0.25	0.5	0.5–2	1–16
*A. niger*	0.25	0.5	0.5–2	2–4
*A. terreus*	0.25	0.25	0.5–1	0.5–4
*Blastomyces* spp.	0.063	0.125	1	-
*Histoplasma* spp.	0.019	0.25	0.5	2
*Coccidioides* spp.	0.125	0.25	0.25	0.5
*Fusarium* spp.	16	32	8–>16	>8–>16
*Rhizopus* spp.	1	8	0.25–4	1–>8
*Mucor* spp.	1	16	1–>8	2–>8
*Scedosporium apiospermum*	0.25	1	1–2	2–4
*Scedosporium prolificans*	16	32	>16	-

## 5. Clinical Efficacy

### 5.1. Posaconazole

All posaconazole formulations are approved for the prophylaxis of invasive aspergillosis and candidiasis among high risk patients (e.g., hematopoietic stem cell transplant recipients with graft-versus-host disease (GVHD) or those with hematologic malignancies with prolonged neutropenia from chemotherapy). The posaconazole oral suspension is also FDA-approved for the treatment of oropharyngeal candidiasis, including cases refractory to itraconazole and/or fluconazole [11]. However, posaconazole has been increasingly used for unlabeled indications. Use of posaconazole for these indications has been primarily supported by smaller, non-randomized studies utilizing the posaconazole suspension. As the intravenous and delayed-tablet formulations achieve greater serum concentrations, equivalent or improved efficacy for these unlabeled indications is expected and utility of these newer formulations is extrapolated from previously published literature. Hence, studies utilizing the posaconazole suspension will be reviewed.

Currently, posaconazole is recommended as a first line agent for antifungal prophylaxis for neutropenic patients with myelodysplasia (MDS), acute myeloid leukemia (AML), and patients with significant GVHD. [30] Data supporting these recommendations originate from two randomized trials in which the posaconazole oral suspension was compared to fluconazole or itraconazole [31,32]. A randomized, multicenter study evaluated 602 chemotherapy-induced neutropenic patients with acute myeloid leukemia (AML) or myelodysplasia (MDS) [30]. Patients were randomly assigned to receive posaconazole 200 mg oral suspension three times daily (*n* = 304) or either fluconazole 400 mg oral suspension daily (*n* = 240) or itraconazole 200 mg oral solution twice daily (*n* = 58). The primary endpoint was the incidence of proven or probable IFI, according to the European Organization for the Research and Treatment of Cancer and the Mycoses Study Group (EORTC/MSG) criteria [33]. Significantly fewer patients in the posaconazole group, compared to the fluconazole or itraconazole group, developed proven or probable IFI during the treatment phase (2% *vs.* 8%, *p* < 0.001), including significantly fewer cases of aspergillosis associated with posaconazole prophylaxis than with fluconazole or itraconazole prophylaxis (1% *vs.* 7%, *p* < 0.001). A subsequent *post hoc* analysis limited to centers where fluconazole was the comparator, demonstrated superiority of posaconazole compared to fluconazole for the prevention of proven or probable IFI (2% *vs.* 9%, *p* = 0.001 during the treatment period; 3% *vs.* 11%, *p* = 0.001 at 100 days). Additionally, a Kaplan-Meier analysis of time to all-cause mortality demonstrated a survival benefit among patients receiving posaconazole compared to those receiving fluconazole or itraconazole (*p* = 0.04). A randomized, multicenter, double-blind trial compared posaconazole 200 mg oral suspension three times daily to fluconazole 400 mg oral capsule once daily for an expected 112-day fixed treatment period among 600 HSCT patients with GVHD who had received intensive immunosuppressive therapy [31]. Posaconazole was found to be as effective as fluconazole for the prevention of proven or probable IFI (5.3% *vs.* 9.0%, respectively; odds ratio, 0.56; 95% confidence interval [CI], 0.30 to 1.07); and it was found to be superior to fluconazole for the prevention of proven or probable invasive aspergillosis (2.3% *vs.* 7.0%, *p* = 0.006). Overall mortality was similar in the two groups; however there were fewer deaths caused by IFI in the posaconazole group (1%, *vs.* 4%, *p* = 0.046).

The new posaconazole formulations have been studied for the prevention of IFI in two non-randomized trials. A phase III, multicenter, open-label study evaluated the posaconazole delayed-release tablet for the prevention of proven or probable IFI among 210 patients with neutropenia, AML, MDS, and post-allogeneic HSCTs. All patients received posaconazole delayed-release tablet 300 mg orally daily. The average steady state posaconazole concentration among PK-evaluable patients (*n* = 50) was 1580 ng/mL. Efficacy endpoints included the incidence of proven or probable IFI (one patient with *C. glabrata* infection of the pleura) and survival at 65 days (192/210, 91%) [20]. A phase III, multicenter, open-label study of IV posaconazole evaluated 237 neutropenic patients with AML, MDS, and post-allogeneic HSCT. Patients received posaconazole 300 mg IV BID on day one, followed by 4–13 days of posaconazole IV once daily, then posaconazole oral suspension 400 mg twice daily or 200 mg three times daily to complete a total of 28 days. Among 49 PK-evaluable patients, the average steady state posaconazole serum concentration achieved from 300 mg IV once daily was 1500 ng/mL and 94% of patients achieved the pre-specified posaconazole exposure target of steady-state plasma average serum posaconazole concentration between 500 and 2500 ng/mL. Only three (1%) cases of proven or probable IFI were reported during the study period [34].

Two studies have evaluated the use of posaconazole for oropharyngeal and esophageal candidiasis. A multicenter, randomized trial compared posaconazole oral suspension to fluconazole for the treatment of oropharyngeal candidiasis (*n* = 350) in patients with human immunodeficiency virus (HIV). Patients were randomized to receive posaconazole oral suspension or fluconazole oral suspension for a total of 14 days. On day 14, patients receiving posaconazole achieved similar rates of clinical and mycologic success as those receiving fluconazole. By day 42, significantly more patients receiving posaconazole continued to have mycologic success (40.6% *vs.* 26.4%, *p* = 0.038) [35]. A phase III, multicenter, open-label study, evaluated two dosing regimens of posaconazole oral suspension among 176 HIV-infected patients with fluconazole- and itraconazole-refractory oropharyngeal and esophageal candidiasis. Both dosing regimens resulted in a 75% rate of cure or improvement at the end of treatment [36]. While fluconazole remains the first line systemic antifungal agent for the treatment of oropharyngeal and esophageal candidiasis, posaconazole is an alternative option for the treatment of refractory disease [37].

Voriconazole and amphotericin B remain the first and second line agents for the treatment of invasive aspergillosis, respectively. Still, one study has described success utilizing posaconazole for the treatment of invasive aspergillosis among patients who were refractory or intolerant of conventional therapy. The majority of patients had a hematological malignancy (*n* = 79). Additionally, 48 patients had a history of allogeneic HSCT and 12 patients had a history of SOT. Sites of aspergillosis infection included pulmonary (*n* = 79), extrapulmonary (*n* = 19), and disseminated (*n* = 4). Posaconazole oral suspension 800 mg/day in divided doses was administered as monotherapy to 107 patients with confirmed invasive aspergillosis who failed to improve after at least seven days of antifungal therapy (*n* = 94) or were intolerant of conventional therapy (*n* = 13). The most common prior antifungal therapy received by study subjects was conventional amphotericin B, a lipid formulation of amphotericin B, and/or itraconazole. These patients were compared to 86 retrospective control patients who received the institution-specific standard of care for salvage therapy. More patients receiving posaconazole had a successful global response compared to the control group (42% *vs.* 26%, *p* = 0.005). Additionally, significantly more patients treated with posaconazole survived at the end of therapy compared to control subjects (38% *vs.* 22%, *p* = 0.0003) [38]. A double-blind, randomized study evaluating the safety and efficacy voriconazole compared to the new posaconazole formulations (intravenous loading dose and delayed-release tablet maintenance dose) for the treatment of aspergillosis is currently recruiting.

Limited options are available for the treatment of mucormycosis. Surgery, whenever possible, is generally recommended combined with antifungal treatment. Liposomal amphotericin B is currently \the drug of choice for mucormycosis and posaconazole is an option for salvage therapy [39]. Two retrospective studies have described the use of posaconazole suspension for the treatment of mucormycosis refractory or intolerant to standard therapy. A case-series described 24 patients with active proven, probable, or possible mucormycosis who were refractory (*n* = 19) or intolerant (*n* = 5) to standard therapy. The majority of patients had hematologic malignancies treated with allogeneic bone marrow transplant or allogeneic peripheral-blood stem cell transplant (*n* = 11). Other common risk factors included SOT (*n* = 4) and diabetes mellitus (*n* = 5). Patients were treated with posaconazole oral suspension 800 mg daily in two or four divided doses. The most common site of infection was rhinocerebral (46%) and 33% of all patients underwent surgical debridement. Overall, 79% of patients had a complete or partial response, based on clinical and radiologic improvement (if available). Surgery was marginally associated with a reduced risk of clinical failure (hazard rate, 0.17; 95% CI, 0.03–1.07). Overall mortality was 37.5% (9/24); 16.7% (4/24) was directly attributed to mucormycosis [40]. The second study, was a retrospective analysis of 91 patients with proven (*n* = 69) or probable (*n* = 22) mucormycosis who were refractory (*n* = 48) or intolerant (*n* = 10) to standard therapy, or both (*n* = 33). The most common predisposing conditions were hematologic malignancies (*n* = 48), diabetes mellitus (*n* = 30), receipt of chronic steroid treatment (*n* = 31), and GVHD (*n* = 30). Patients received posaconazole oral suspension 800 mg daily divided in two or four divided doses. The most common sites of infection were the sinuses (46%) and pulmonary (41%). Successful (complete or partial) response at 12 weeks was observed in 60% of patients. Thirteen heavily immunosuppressed patients received greater than one week of posaconazole in combination with lipid formulations of amphotericin B. Among patients receiving combination therapy, 46% had a partial response, 23% had stable disease, and 31% had failure. The success rate was also similar among patients who did and did not receive adjunctive surgical procedures (61% *vs.* 62%) [41].

### 5.2. Isavuconazole

Three phase III programs are currently under development to examine the safety and efficacy of isavuconazole for the treatment of IFI. Based on results from the SECURE and VITAL studies, isavuconazole was recently approved for the treatment of invasive aspergillosis and mucormycosis. Recruitment for the ACTIVE trial evaluating isavuconazole for the treatment of candidiasis has been completed, but results are not anticipated until the end of 2015.

The SECURE study was a multinational, randomized, double-blind, non-inferiority trial evaluating the safety and efficacy of isavuconazole *vs.* voriconazole in the primary treatment of IFI caused by *Aspergillus* spp. and other filamentous fungi [42]. Patients with proven, probable, or possible disease based on EORTC criteria were randomized 1:1 to receive either isavuconazole 200 mg IV every 8 h for two days followed by 200 mg daily orally or IV (*n* = 258) or voriconazole 6 mg/kg IV twice daily followed by 4 mg/kg twice daily (*n* = 258) up to 84 days. Starting on the third day of treatment, patients randomized to voriconazole could be switched to voriconazole orally 200 mg twice daily. The primary efficacy endpoint was 42-day all-cause mortality. The major secondary endpoint was overall response at the end-of-treatment in patients with proven or probable IFI. The majority of patients had invasive aspergillosis (85%) with pulmonary involvement (92%). Most patients had an underlying hematologic malignancy (84%) and were neutropenic (65%), while 20% of patients had a prior allogeneic HSCT. Non-inferiority was demonstrated for the primary endpoint of 42-day all-cause mortality in the intent-to-treat (ITT) population (isavuconazole 18.6%, voriconazole 20.2%; absolute difference −1.6; 95% CI, −7.8%, 5.7%). Overall response rates at the end-of-treatment were 35.0% and 36.4% for isavuconazole and voriconazole, respectively [41]. Despite less than half of subjects having proven (7%) or probable (38%) infection in the ITT population, the rates of mortality and overall response did not differ between isavuconazole and voriconazole in both the overall and ITT analyses. Due to the limited clinical experience with isavuconazole, voriconazole will likely remain as the drug of choice for invasive aspergillosis. However, based on these data, isavuconazole may serve as a viable option in patients intolerant of voriconazole.

The VITAL study was an open-label, multicenter study evaluating the safety and efficacy of isavuconazole for the treatment of invasive aspergillosis in patients with renal impairment and in IFI caused by rare fungi, including mucormycosis [43]. Patients were treated for proven or probable IFI according to EORTC/MSG criteria, including those that required primary therapy or those that were refractory or intolerant to prior antifungal therapy. Isavuconazole 200 mg IV every 8 h was given for two days followed by 200 mg daily orally or IV for up to 180 days. The primary endpoint was overall response at the end of treatment based on clinical, mycological, and radiographical criteria as determined by an independent data review committee (DRC). Successful overall response consisted of either complete or partial response. Secondary endpoints included 42-day and 84-day all-cause mortality. Of the 149 patients enrolled in the VITAL trial, 37 patients were determined to have proven (*n* = 32) or probable (*n* = 5) invasive mucormycosis. Of these 37 patients, 24 patients discontinued the study due to death (*n* = 11), adverse event or intercurrent illness (*n* = 6), insufficient therapeutic response (*n* = 2), or other reasons (*n* = 5). The majority of patients had pulmonary disease (*n* = 22). The most common sites of non-pulmonary disease were sinus (*n* = 16), eye (*n* = 7), and central nervous system (*n* = 6); 32% of patients had involvement of multiple sites. The most common primary underlying diseases were acute myeloid leukemia (*n* = 10), diabetes mellitus (*n* = 4), and acute lymphocytic leukemia (*n* = 3) with 13 patients having allogeneic HSCT. At the end of treatment, the DRC determined that 11 (31%) patients had a successful overall response. Mortality at 42 days was 38% and 43% at 84 days [43]. Compared with previous studies, these rates are similar to studies evaluating the use of amphotericin B (overall mortality 38%–55%) and posaconazole (overall mortality 38%, attributable mortality 16%–17%) in the treatment of invasive mucormycosis [40,41,44,45,46].

The VITAL study also included nine patients treated for cryptococcosis [47]. Isolated pathogens were *C. neoformans* (*n* = 4) and *C. gattii* (*n* = 3). Three patients had isolated pulmonary disease and two patients had central nervous system disease. The remaining patients had disseminated disease in the lung, central nervous system, and blood or other organs. At the end of treatment, six patients were considered treatment successes, and eight patients were alive through 84 days.

The ACTIVE study is a randomized, double-blind, phase III study comparing the safety and efficacy of isavuconazole to caspofungin followed by voriconazole for up to 56 days for the treatment of invasive candidiasis including candidemia [48]. The primary outcome is the overall response at the end of IV therapy as determined by an independent DRC based on resolution of signs and symptoms and mycological eradication. Data for the ACTIVE study are anticipated by late 2015. However, given the established efficacy and safety of fluconazole and echinocandins in the treatment of invasive candidiasis, isavuconazole is unlikely to displace these agents as the antifungals of choice for infections due to *Candida* spp.

## 6. Adverse Effects

### 6.1. Posaconazole

All posaconazole formulations appear to be well-tolerated. Initial phase I studies among healthy volunteers, prompted concerns regarding the potential for increased adverse effects with the posaconazole tablets due to greater posaconazole exposure. Among patients treated with posaconazole delayed-release tablets 200 mg or 400 mg once daily, the most commonly reported treatment-related adverse event was hepatic enzyme elevation (24%) [21]. However, phase III trials of both the delayed-release tablet and IV formulations have not reproduced this increased incidence of elevated hepatic enzymes and similar safety profiles to posaconazole oral suspension have been observed. The incidence of increased hepatic enzymes with standard doses of the posaconazole delayed-release tablet was 4% [34]. Additionally, a study among 12 leukemic patients demonstrated that a transition from posaconazole suspension to delayed-release tablets significantly increased posaconazole serum concentrations, without resulting in a corresponding increase in hepatotoxicity [49]. A phase III trial of the delayed-release tablet described the most common treatment-related adverse events as nausea (11%) and diarrhea (8%). Adverse events led to discontinuation of the delayed-release tablet among 18% of patients, with the most common reason being due to nausea (2%) [20]. Similarly, diarrhea (8%), nausea (5%), and rash (5%) were the most commonly reported treatment-related adverse events with the IV formulation [34]. As the use of the new posaconazole formulations increases, further monitoring is warranted to identify any additional adverse events.

### 6.2. Isavuconazole

Based on data from phase I and phase II trials, isavuconazole has a favorable safety profile [16,17,25]. In healthy adults, no serious adverse events in a dose-ascending study from 50 to 400 mg were seen. Some of the most frequent side effects reported were mild upper abdominal pain, headache, nasopharyngitis, and rhinitis. In two phase II studies, drug-related adverse events occurred in 54% of neutropenic patients receiving isavuconazole as antifungal prophylaxis (*n* = 24) and 18% of patients receiving isavuconazole for treatment of esophageal candidiasis (*n* = 121). Discontinuation of isavuconazole occurred in four patients in each of the two studies [50,51].

In the SECURE trial, treatment-emergent adverse events occurred in 96% of isavuconazole patients and 99% of voriconazole patients, but drug-related adverse events occurred in 42% and 60% of patients, respectively [42]. The most common adverse events reported were nausea, vomiting, pyrexia, and diarrhea and were similar between both groups. Drug-related adverse events were reported in 42% of isavuconazole-treated subjects and 60% of voriconazole-treated patients. Significantly fewer adverse events were reported in the isavuconazole treatment group in the system organ classes of skin (34% *vs.* 44%), eye (15% *vs.* 27%), and hepatobiliary disorders (9% *vs.* 16%). In the VITAL study, treatment-emergent adverse events and drug-related adverse events were reported in 35 (95%) of patients and 13 (35%), respectively, in patients with invasive mucormycosis [47]. Three (8%) patients had serious drug-related adverse events.

## 7. Drug Interactions

### 7.1. Posaconazole

Medications affecting gastrointestinal pH and motility are known to affect posaconazole oral suspension. However, no clinically relevant effects on the pharmacokinetics were observed with posaconazole delayed-release tablets when concomitantly used with antacids, H_2_-receptor antagonists, proton pump inhibitors, and gastric motility agents [52]. The stability of the delayed-release tablet under these conditions may be especially beneficial among SOT patients whom are often prescribed gastric acid suppression therapy.

Posaconazole is a strong inhibitor of the hepatic and extrahepatic cytochrome P450 CYP3A4 enzyme. CYP3A4 activity displays large inter-individual variability. Inhibition of said enzyme can result in increased plasma concentrations of medications metabolized by CYP3A4. However, the extent of this increase is often difficult to predict [53]. Co-administration of these agents should be closely monitored, as increased side effects and toxicity may result, especially given the increased serum concentrations obtained by the newer posaconazole formulations [11]. In addition, due to posaconazole hepatic metabolism by UDP glucuronidation, inducers of UDP glucuronidation, such as rifabutin and phenytoin have been associated with significant reductions in posaconazole concentration.

Importantly, concomitant use with tacrolimus, cyclosporine, and sirolimus may result in increased concentrations of these immunosuppressive agents, as they are CYP3A4 and P-gp substrates. In a study performed among heart transplant recipients, posaconazole suspension (400 mg twice daily for eight days) was reported to increase the area under the concentration-time curve (AUC) for tacrolimus by 358%. Additionally, posaconazole suspension (200 mg twice daily for 10 days) prompted a 14%–29% cyclosporine dose reduction. Dose reductions of tacrolimus and cyclosporine should occur at the initiation of posaconazole therapy and serum concentrations should be frequently monitored [54]. Concomitant therapy with sirolimus should be avoided, as approximately nine-fold increases in sirolimus AUC have been observed, resulting in an increased risk of sirolimus toxicity [11,20,55]. Greater CYP3A4 enzyme inhibition may be possible with the posaconazole delayed-release tablet and intravenous formulations due achievement of higher serum concentrations. Careful monitoring should occur when initiating and discontinuing posaconazole therapy. The major drug-drug interactions with posaconazole are summarized in Table 3.

**Table 3:** Summary of major drug-drug interactions involving posaconazole and isavuconazole.

Coadministered Drug	Posaconazole	Isavuconazole
*Immunosuppressants*		
Sirolimus	Contraindicated	Use with caution (increased sirolimus concentrations)
Tacrolimus	Reduce tacrolimus dose to one-third the original dose upon initiation of posaconazole	Use with caution (increased tacrolimus concentrations)
Cyclosporine	Reduce cyclosporine dose to three-fourths of original dose upon initiation of posaconazole	Use with caution (increased cyclosporine concentrations)
Mycophenolate	-	Use with caution (increased mycophenolate concentrations)
*Antiretrovirals*		
Efavirenz	Avoid combination (decreased posaconazole concentrations)	-
Ritonavir	Monitor for toxicities (increased ritonavir concentrations)	-
Atazanavir	Monitor for toxicities (increased atazanavir concentrations)	-
Fosamprenavir	Monitor for breakthrough fungal infections (decreased posaconazole concentrations)	-
Lopinavir/ritonavir	-	Use with caution (increased isavuconazole and decreased lopinavir/ritonavir concentrations)
*Gastrointestinal agents*		
Metoclopramide	Monitor for breakthrough fungal infections (decreased posaconazole concentrations) ^#^	-
Cimetidine	Monitor for breakthrough fungal infections (decreased posaconazole concentrations) ^#^	-
Esomeprazole	Monitor for breakthrough fungal infections (decreased posaconazole concentrations) ^#^	-
*Other*		
Rifampin	-	Contraindicated
Rifabutin	Avoid combination (increased rifabutin and decreased posaconazole concentrations)	-
Ketoconazole	-	Contraindicated
Phenytoin	Monitor for phenytoin toxicity and breakthrough fungal infections (increased phenytoin and decreased posaconazole concentrations)	-
Buproprion	-	Consider dose increase of buproprion (decreased concentrations)
Vinca alkaloids	Consider dose reduction of vinca alkaloids and monitor for toxicities (increased concentrations)	-
Calcium channel blockers metabolized by CYP3A4 (e.g., verapamil, diltiazem)	Monitor for toxicities (increased calcium channel blocker concentrations)	-
Digoxin	Monitor digoxin concentrations and titrate dose (increased digoxin concentrations)	Monitor digoxin concentrations and titrate dose (increased digoxin concentrations)
Simvastatin	Contraindicated	-
Atorvastatin	Use with caution (potential for increased atorvastatin concentrations)	Use with caution (potential for increased atorvastatin concentrations)
Ergot alkaloids	Contraindicated	-
Benzodiazepines metabolized by CYP3A4 (e.g., midazolam, alprazolam)	Monitor for benzodiazepine adverse effects and consider dose reduction	Monitor for benzodiazepine adverse effects and consider dose reduction

^#^ Only when given with posaconazole oral suspension due to decreased absorption. No significant effects when given with posaconazole tablets.

### 7.2. Isavuconazole

Although food and pH effects have no bearing on isavuconazole concentrations, it has significant potential for drug-drug interactions since it is primarily metabolized and is a moderate inhibitor of CYP3A4. Data regarding disposition of isavuconazole and interacting drugs is still limited. Some studies have evaluated interactions between isavuconazole and major inducers, inhibitors, and substrates of the CYP3A4 pathway. Co-administration of isavuconazole and rifampin, a strong inducer of CYP3A4, resulted in a fourfold decrease in Cmax and a 40-fold decrease in AUC whereas ketoconazole, a strong inhibitor of CYP3A4, increased Cmax by 9% and AUC by 422% [56,57]. Co-administration of isavuconazole with strong inducers and inhibitors of CYP3A4 is contraindicated. Isavuconazole should be used with caution with mild or moderate inducers and inhibitors of CYP3A4.

Tacrolimus exposure was increased by 75% in healthy subjects concurrently receiving isavuconazole [58]. On the contrary, pharmacokinetic studies of the impact of isavuconazole on cyclosporine and warfarin concentrations demonstrated no significant changes in their pharmacokinetics [59,60]. These studies highlight the need for additional studies to directly examine the impact of isavuconazole on the pharmacokinetics of other drugs metabolized through the CYP3A4 system. In the absence of this data, potential drug-drug interactions should be predicated on moderate inhibition of this pathway by isavuconazole. Other drug interaction studies regarding the impact of isavuconazole on other drugs is summarized in Table 3.

## 8. Therapeutic Drug Monitoring

### 8.1. Posaconazole

Evidence describing the exposure-response relationship is limited and somewhat controversial. In attempts to correlate pharmacokinetic data with the prophylactic efficacy of posaconazole, data from two clinical trials [31,32] were compiled [61]. An inverse relationship between average steady state concentrations (Cavg) and clinical failure was observed, wherein patients with higher Cavg had reduced rates of clinical failure [61]. However, critics remark that the study’s use of a composite definition of clinical success, no premature discontinuation, no invasive fungal disease, and no death, may limit the clinical significance of this correlation [62]. Subsequent studies with more robust outcome parameters (e.g., incidence of breakthrough IFI) have supported an exposure-response relationship with posaconazole [63,64]. Still, therapeutic drug monitoring has become common practice with posaconazole, primarily due to the variable absorption of the posaconazole suspension. The majority of studies examining the use of posaconazole for prophylaxis suggest a serum concentration greater than 500–700 ng/mL for clinical success [31,32,63,65]. Therapeutic serum concentration recommendations for the treatment of infections are less clear. However, troughs greater than 500–1500 ng/mL and average concentration greater than 1250 ng/mL have been proposed [38,63]. While current literature suggests a role for therapeutic drug monitoring to guide dosing, there are no consensus guidelines to provide recommendations for target concentrations and sample timing. With more reliable absorption among the newer posaconazole formulations, the need for therapeutic drug monitoring may shift in the future. If therapeutic serum concentrations are attained consistently, the need to document therapeutic serum concentrations for efficacy may be reduced. Alternatively, if concentration-related adverse events emerge, an upper serum concentration limit may need to be established in attempts to minimize toxicity. However, previous studies have not identified a clear relationship between toxicity and serum concentrations achieved by the posaconazole suspension [55,61].

### 8.2. Isavuconazole

The utility of therapeutic drug monitoring for isavuconazole has yet to be determined as isavuconazole concentrations have not yet been correlated with either safety or efficacy endpoints. However, several other factors may obviate the need for therapeutic drug monitoring with isavuconazole [66]. These factors include low-to-moderate intra- and interpatient pharmacokinetic variability and an apparent large therapeutic window as demonstrated by the good tolerability profile of isavuconazole. Therapeutic monitoring is not recommended at present.

## 9. Summary and Implications on the Clinical Impact in Transplant Patients

Advances in the treatment and management of patients with hematologic malignancies, bone marrow, and solid organ transplant recipients, and other immunocompromised patients comprised an at-risk population susceptible to IFI which is ever-increasing. However, due to the limited armamentarium of antifungal agents, treatment of these IFIs remains a challenge for health care professionals. Unlike other azole antifungals, both posaconazole and isavuconazole exhibit broader spectrums of activity, most notably against Mucorales. They offer additional therapeutic options for these difficult to treat IFIs.

With consistent and reliable achievement of adequate serum concentrations, even among patients with limited food intake and on acid suppression therapy, the new posaconazole formulations may offer improved clinical success among a variety of therapeutic areas. The role of posaconazole has been firmly established for the prevention of invasive aspergillosis and candidiasis among immunocompromised patients, particularly among hematopoietic stem cell transplant recipients. Compared to the oral suspension, use of the delayed-release tablet for prophylaxis will likely reduce the cost associated with frequent therapeutic drug monitoring and dosage adjustment required to obtain target serum concentrations. Consequently, the delayed-release tablet may replace the oral suspension among patients who are able to take oral therapy. However, the need for the posaconazole suspension will remain among patients in which enteral administration is preferred, as the delayed-release tablets cannot be crushed.

Limited robust clinical data are currently available to support the use of posaconazole for the treatment of IFI. In the past, the use of posaconazole oral suspension may have been limited by the inability to achieve reliable serum concentrations, especially in the acute setting where the desire to rapidly initiate effective therapy exists. However, with trials demonstrating consistent average serum concentrations greater than 1250 ng/mL, the utility of posaconazole for the treatment of IFIs may increase, including among patients with severe infection where intravenous therapy may be preferred. Additionally, among patients with aspergillosis and mucormycosis, posaconazole may offer slightly fewer drug-drug interactions and a milder adverse event profile, compared to the current first-line agents. However, robust clinical trials are still required to demonstrate the use of posaconazole in these areas.

The newly approved antifungal, isavuconazole, is currently indicated for the treatment of both invasive aspergillosis and mucormycosis but is a promising option for other IFIs pending additional clinical data. Pharmacologically, isavuconazole offers several advantages over other azole antifungals. Unlike voriconazole, isavuconazole displays linear pharmacokinetics and its disposition is not impacted by genetic polymorphisms. These factors result in low interpatient variability of isavuconazole concentrations reducing the need for therapeutic drug monitoring, which is currently recommended for most other azole antifungals. Additionally, isavuconazole is formulated as a highly water-soluble prodrug that does not require co-formulation with cyclodextrin eliminating concerns of potential nephrotoxicity associated with the intravenous forms of voriconazole and posaconazole. Other advantageous pharmacologic factors include extensive bioavailability, high volume of distribution, long half-life, and low clearance. However, as with other agents in the azole antifungal class, drug interactions remain an issue with isavuconazole due to inhibition of the cytochrome P450 system.

With the new oral and intravenous formulations, the differences between posaconazole and isavuconazole are not as disparate as compared with the posaconazole oral suspension. Except for a few minor pharmacologic differences, both posaconazole and isavuconazole appear to have a similar place in therapy. Posaconazole has the benefits of fewer anticipated drug interactions and greater clinical experience whereas isavuconazole does not contain cyclodextrin and likely does not require therapeutic drug monitoring. Regardless of these differences, both agents are promising alternatives for aspergillosis and mucormycosis. Fluconazole and the echinocandins will likely retain their major role in the treatment of invasive candidiasis, but additional clinical data are needed for other IFIs.

## 10. Conclusions

Invasive fungal infections are a major cause of morbidity and mortality among SOT and HSCT recipients. Previously available antifungal agents have been limited by their spectrum of activity and pharmacokinetic profiles. Isavuconazole and newer formulations of posaconazole offer additional treatment options for these group of patients. However, further studies are needed to better define their place in therapy.
